# A biologist’s guide to planning and performing quantitative bioimaging experiments

**DOI:** 10.1371/journal.pbio.3002167

**Published:** 2023-06-27

**Authors:** Rebecca A. Senft, Barbara Diaz-Rohrer, Pina Colarusso, Lucy Swift, Nasim Jamali, Helena Jambor, Thomas Pengo, Craig Brideau, Paula Montero Llopis, Virginie Uhlmann, Jason Kirk, Kevin Andrew Gonzales, Peter Bankhead, Edward L. Evans, Kevin W. Eliceiri, Beth A. Cimini

**Affiliations:** 1 Imaging Platform, Broad Institute of MIT and Harvard, Cambridge, Massachusetts, United States of America; 2 Live Cell Imaging Laboratory, University of Calgary, Calgary, Alberta, Canada; 3 National Center for Tumor Diseases, University Cancer Center, NCT-UCC, Universitätsklinikum Carl Gustav Carus an der Technischen Universität Dresden, Dresden, Germany; 4 Informatics Institute, University of Minnesota Twin Cities, Minneapolis, Minnesota, United States of America; 5 MicRoN Core, Harvard Medical School, Boston, Massachusetts, United States of America; 6 European Bioinformatic Institute, European Molecular Biology Laboratory, Wellcome Genome Campus, Hinxton, United Kingdom; 7 Optical Imaging & Vital Microscopy Core, Baylor College of Medicine, Houston, Texas, United States of America; 8 Mammalian Cell Biology and Development, Rockefeller University, New York, New York, United States of America; 9 Edinburgh Pathology, Centre for Genomic and Experimental Medicine, and CRUK Scotland Centre, Institute of Genetics and Cancer, University of Edinburgh, Edinburgh, United Kingdom; 10 Morgridge Institute and University of Wisconsin-Madison, Madison, Wisconsin, United States of America

## Abstract

Technological advancements in biology and microscopy have empowered a transition from bioimaging as an observational method to a quantitative one. However, as biologists are adopting quantitative bioimaging and these experiments become more complex, researchers need additional expertise to carry out this work in a rigorous and reproducible manner. This Essay provides a navigational guide for experimental biologists to aid understanding of quantitative bioimaging from sample preparation through to image acquisition, image analysis, and data interpretation. We discuss the interconnectedness of these steps, and for each, we provide general recommendations, key questions to consider, and links to high-quality open-access resources for further learning. This synthesis of information will empower biologists to plan and execute rigorous quantitative bioimaging experiments efficiently.

## Abstract 2

As bioimaging has moved from an observational method to a quantitative one, additional considerations are needed to carry out this work in a rigorous and reproducible manner. This Essay provides a navigational guide for biologists new to quantitative bioimaging, with recommendations and pointers to open source resources.

## Introduction

The optical microscope is a major cornerstone of biological discovery, yet modern-day bioimaging experiments involve much more than looking down the eye-piece and reporting observations. Proper quantification of imaging data requires planning and decision-making at every step of the experimental imaging workflow.

In practice, even the most motivated and independent learner cannot master the best practices in microscopy if they do not know what topics they must fully understand and which skills they need to acquire as they work towards proficiency. Ideally, biologists wishing to learn more about microscopy will have convenient local access to training and mentorship as they develop their bioimaging knowledge and skills. Unfortunately, in practice, access to advanced training and support varies widely by site. Thankfully, in addition to a number of hands-on specialized workshops that provide excellent training opportunities, a wealth of high-quality open-source resources have been generated by the bioimaging community.

**Fig 1:**
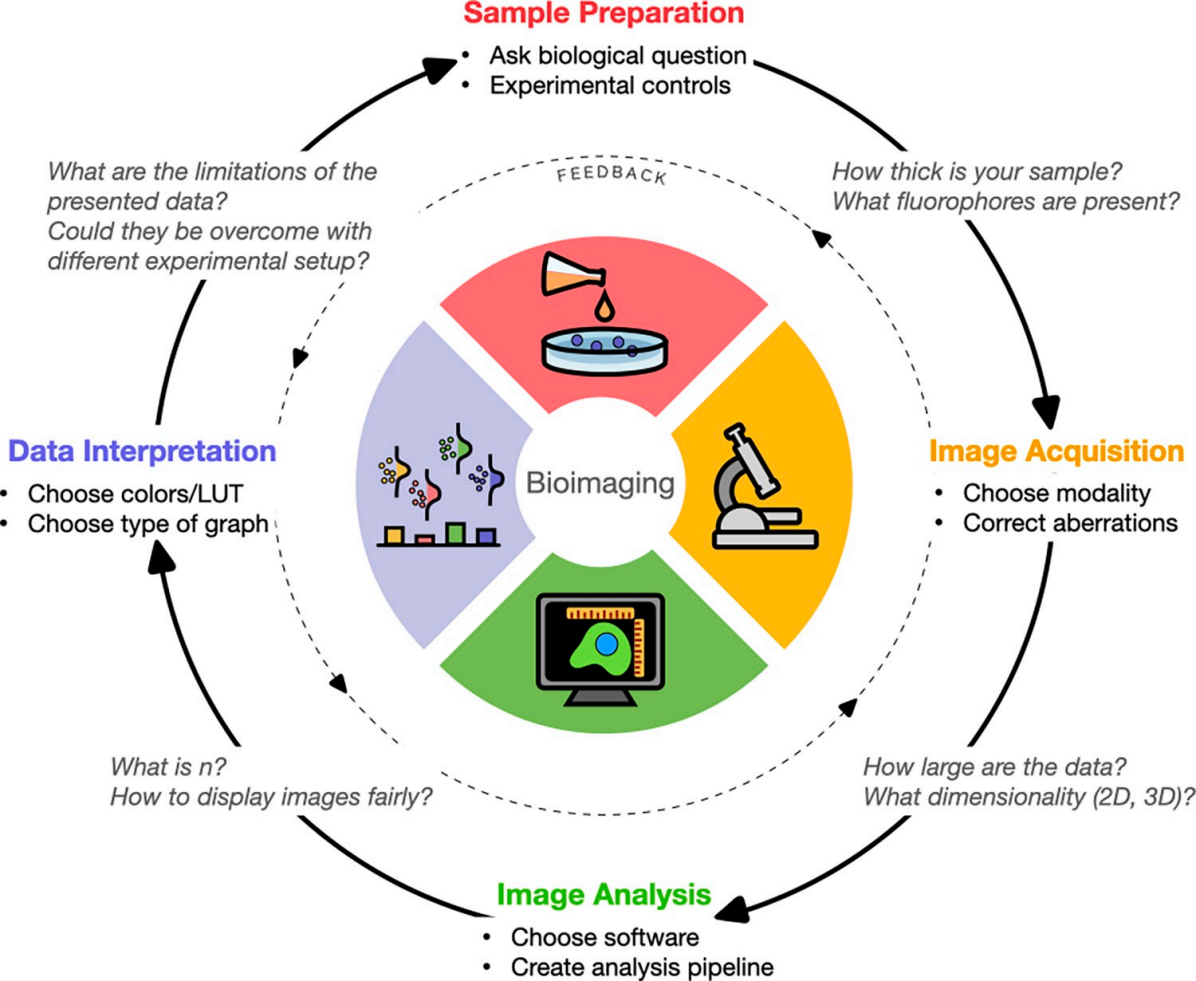
The steps of any quantitative bioimaging experiment are mutually interconnected. Each quadrant represents one of the 4 key elements to any bioimaging experiment, from sample preparation, to image acquisition, image analysis, and data interpretation. Between quadrants, example questions demonstrate how decisions made at one level feed forward to affect subsequent steps. Analogously, desired endpoints inform how earlier steps should be performed. Created with BioRender.com.

This Essay serves as a navigational guide for the important considerations needed to best utilize optical microscopy as a quantitative measurement technique. For each major stage of bioimaging experiments (sample preparation, image acquisition, image analysis, and data interpretation; Fig 1), we present key high-level considerations as well as highlighting resources that present further information. We primarily focus on fluorescence microscopy of animal cells and tissues, but many recommendations and resources are generalizable to other kinds of bioimaging (e.g., brightfield microscopy, electron microscopy, histology) and other sample types (plants, prokaryotes, etc.).

**Fig 2:**
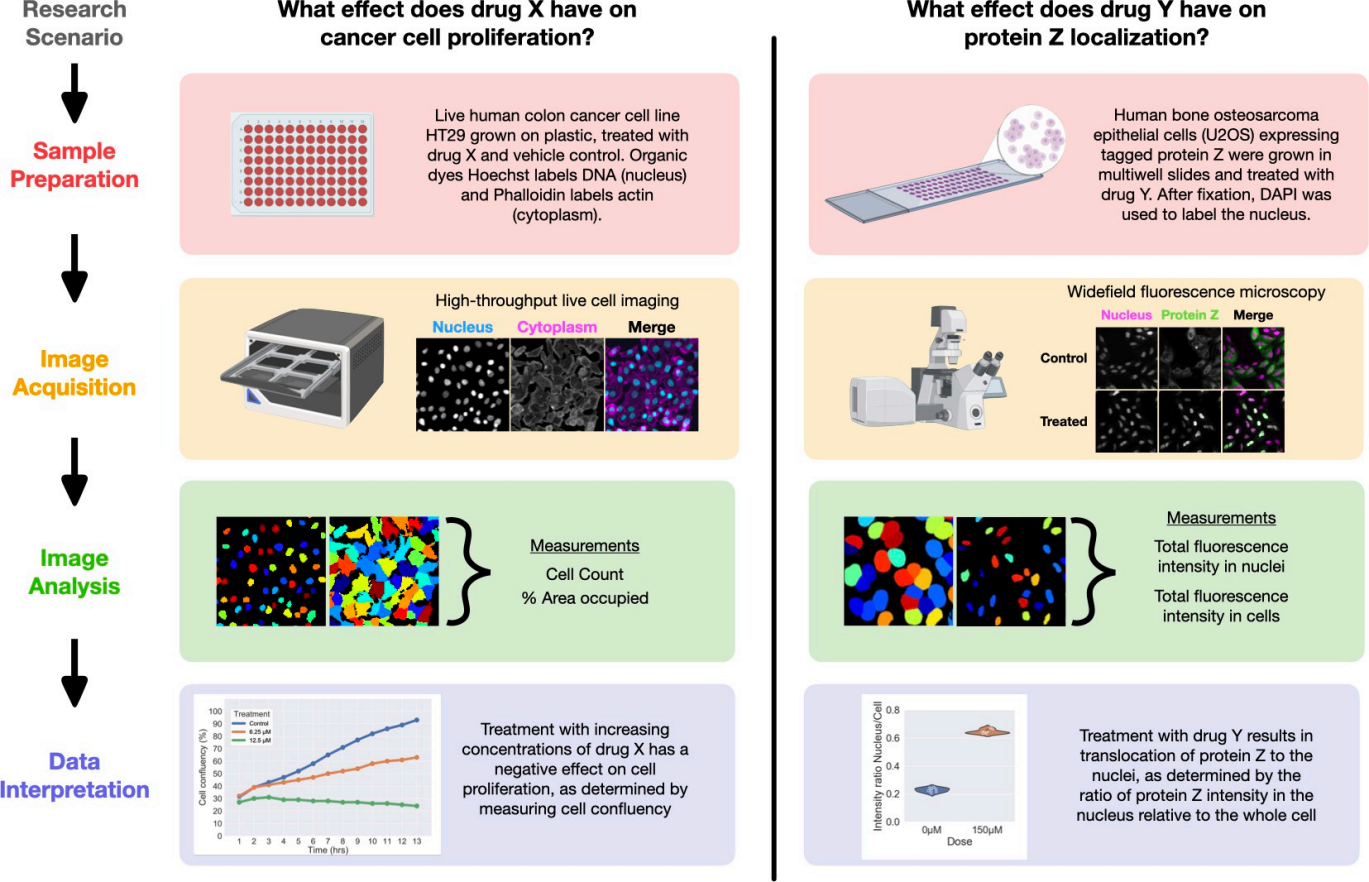
Two quantitative bioimaging example experiments. Each hypothetical scenario is broken down into the 4 major stages of quantitative bioimaging and demonstrates how a biological question is translated into an experiment that yields interpretable data. Images are sourced from BBBC008v1 [1] and BBBC013v1 (provided by Ilya Ravkin) available from the Broad Bioimage Benchmark Collection [1,2]. Created with BioRender.com.

Throughout these sections, a few recurring principles can be identified. First, there is no single correct answer for questions in quantitative bioimaging. In nearly all aspects of a microscopy experiment, there are many possible options; this is because the optimal choice for a particular set of conditions depends on the goal of the experiment, the details of the system and sample, and many other factors. We have provided a checklist (Box 1) of key questions to consider when embarking on a quantitative bioimaging experiment. Second, speak to experts before beginning. These include microscopy hardware experts, imaging scientists, image analysts, statisticians, and other biologists. Such experts may be found locally in institutional core facilities or online via community-wide forums. Third, the major steps of bioimaging experiments are highly interconnected (Fig 1). Decisions at one stage affect what is possible at others. It helps to think about experimental design not as a linear process but as a “reverse workflow” in which the desired end result informs the necessary experimental steps and controls; one must always begin experiments with the end in mind. The cycle of a quantitative bioimaging experiment may be repeated multiple times in a project. Pilot experiments can and should be leveraged to quickly test all aspects of a workflow; in a similar fashion, previously published data can also be used as a stepping stone to improve upon existing workflows. Fourth and finally, microscopy is inherently quantitative. Microscopy produces beautiful images that sometimes make it easy to forget that they contain meaningful measurements. Measurements like intensity, area, shape, localization, and many others can provide insight to solve a biologist’s experimental question. By designing rigorous, reproducible experiments with proper controls and optimized workflows, biologists can generate the highest quality data that will give them the power to derive more meaningful conclusions about biology. We have also provided 2 example experiments (Fig 2) to demonstrate how a biological question is translated through the major steps of quantitative bioimaging to generate data and a biological conclusion.

**Box 1. Checklist of key questions when designing bioimaging experiments**

The following list provides questions to consider before embarking on a quantitative bioimaging experiment.

**Sample preparation**

- What is the most appropriate sample type for me to use—cultured cells, tissue sections, whole organisms?
- What samples and/or conditions will I test, including positive and negative controls?
- What dyes or stains (if any) will I use?
- Is my fluorophore combination appropriate?
- What controls must I perform to assess the health of my sample?
- What controls must I perform to address the specificity of my reagents?

**Image acquisition**

- What microscope modality should I use? Do I need confocal?
- Is the microscope modality appropriate for my sample type?
- Am I using the right objective for my sample?
- Are the filter sets appropriate for my fluorophore combination?
- Are my acquisition settings appropriate and consistent?

**Image analysis**

- Have I determined the correct metric for what I want to measure in my images?
- Have I set up my analysis correctly to generate quantitative data?
- Are my measurements made equivalently for controls and experimental samples?
- Are all code, scripts, pipelines, etc. included with my publication materials?
- Is all of the raw data accessible or included with the publication?

**Data interpretation**

- Do qualitative figures comply with best practices on colors used, annotations, and other adjustments?
- Do all microscopy images include a scale bar?
- Have all adjustments made to qualitative figures been disclosed in compliance with best practices and journal policies?
- Have appropriate summary statistics been used for quantitative figures?
- Do my quantitative figures allow the reader to assess the shape of the distribution, including individual plot points wherever possible?

For those looking for more specific recommendations after reading this article, we have used Jupyter Book [3] to create a beginner-friendly interactive companion website. Within, we discuss additional concepts and tips for each section and link to additional high-quality, open-source resources providing in-depth information on specialized topics. By partnering our high-level overview with a more detailed online resource, we aim to provide an on-ramp for a biologist’s first bioimaging experiment as well as a path to sustained learning in the bioimaging community.

## Sample preparation

### General

A high-quality imaging experiment begins at the bench. The best performing microscope will not produce good quality data unless sample preparation is optimized first. Optimal sample preparation helps control for optical aberrations during the microscopy and enables more accurate measurements. When comparing multiple samples, samples should be processed in parallel whenever possible, using the same reagents to avoid technical variability. Positive and negative controls should always be included to ensure that sample processing went as expected and did not introduce artifacts to the imaging [4].

Some aspects of sample preparation are generalizable; major decisions can be roughly divided by preparations designed to be imaged through a coverslip versus preparations where the objective is in direct contact with the sample itself. Most standard applications require imaging through a coverslip, and for most high-resolution imaging experiments, glass thickness should be 0.17 mm (or #1.5 grade coverslips). Using a different thickness does not mean the experiment will not work, but since most objective lenses are corrected for this specific thickness, the image of the sample is brighter, appears sharper, and has better resolution and contrast. It is also critical to choose an appropriate immersion (e.g., air, oil, or water) for the sample holder, objective, and application. All major microscope manufacturing companies make one or more varieties of their own immersion oil. Each oil is optimized for the company’s lenses and sometimes further optimized for specific uses like brightfield or fluorescence imaging. In contrast to approaches utilizing coverslips, microscopy techniques such as tissue and intravital imaging as well as lattice light sheet microscopy (Box 2) use dipping objectives where the objective is partially submerged with the sample. This removes concerns about glass thickness and immersion between glass, but adds considerations about the composition and refractive index (RI) (Box 2) of the sample buffer.

**Box 2. Glossary**

**Lattice light sheet microscopy**

A type of advanced microscope utilizing laser “sheets” of light (in the specific case of lattice light sheet microscopy, optical lattices) that are introduced perpendicular to the imaging path. These approaches can image at higher resolution and speed while minimizing the total amount of light the sample is hit with and thus reducing photobleaching and phototoxicity (see below) in live samples.

**Refractive index**

The measure of how light travels through a particular medium (such as air, water, glass, or the sample itself); changes in refractive index cause bending of light rays traveling through the sample. One typically wishes to minimize refractive index transitions in a given optical setup.

**Phototoxicity**

The loss-of-health or death of a living sample due to damage caused from fluorescent imaging; this commonly is caused by damage created by reactive oxygen species (ROS) created in the fluorescence process.

**Photobleaching**

The gradual loss of fluorescent signal due to irreversible destruction of the fluorophore: typically due to chemical alterations in response to the light used to illuminate the sample.

**Autofluorescence**

Fluorescence signal that the researcher did not intentionally introduce and does not intend to measure, due to intrinsic fluorescent properties of other components in the system (such as molecules in the medium or the plate the sample is contained in). Bright autofluorescence can make measurement of dim “true” signal difficult to impossible.

**Secondary antibody**

Most commonly, immunostaining is done in 2 separate stages: first, the addition of a “primary” antibody against a target the researcher is interested in specifically identifying, and a “secondary” antibody labeled with a fluorophore and targeted to the species of antibody used in the first round. For example, a researcher studying Protein X in mice might use a primary antibody to Protein X made in rabbits, which would be referred to as rabbit-anti-Protein X, followed by an antibody made in goats against the non-sequence-specific sections of rabbit antibodies, which would be referred to as goat-anti-rabbit (plus the name of the dye used). This two-stage procedure has 2 main advantages: the ability to amplify signal (since each primary antibody can be bound by multiple secondary antibodies, meaning more fluorophores are ultimately recruited to each Protein X location) and the ability to not have to attach fluorophores to the huge spectrum of antibodies against primary “targets,” but rather only to the much smaller set of cross-species-antibody antibodies.

**Blocking**

The addition of substances such as gelatin or normal serum to a fixed sample before adding antibodies in order to prevent nonspecific antibody binding.

**Antigen**

The molecular structure an antibody is designed to bind to; sometimes also referred to as an epitope.

**Köhler illumination**

A method of sample illumination that allows for maximal sample brightness and contrast without simultaneously visualizing the light source. The optical components of the microscope must be precisely aligned to set up Köhler illumination, and doing so is critical to the use of phase contrast and differential interference contrast (DIC) microscopy.

**Spherical aberration**

Aberrations that come from spherical lenses refracting light differently near their centers versus near their edges. While modern high-quality objectives contain elements that minimize spherical aberrations, computational methods can sometimes be applied to correct for remaining aberration.

A general principle related to fluorescence imaging is that when selecting fluorophores, one must keep in mind compatibility with the specimen, available illumination sources and filters, and the overall sample preparation workflow. In general, bright and photostable fluorophores are critical, especially when doing live-cell imaging, as their use minimizes phototoxicity and photobleaching (Box 2), which can affect reproducibility in live-cell imaging. This is especially true in prokaryotic organisms, as bio-molecules tend to be present in lower copy number than in eukaryotic cells, and, therefore, bright fluorescent proteins that are monomeric, and thus have less propensity to oligomerize (which can cause localization artifacts), are essential for imaging. Alternatively, small peptide tags that bind to a fluorogenic or fluorescent substrate (usually conjugated with an organic dye), such as SNAP [5] or HALO [6] tags, can be especially useful to image prokaryotes. In general, organic dyes are brighter and more photostable than fluorescent proteins but generally cannot be genetically encoded. If performing multicolor imaging, pay attention to whether the spectra of the different fluorophores overlap [7].

### Live samples

The health of a living sample should always be prioritized, especially as stress is cumulative. As mentioned above, imaging typically requires glass as the sample interface, but many cell types grow poorly when grown directly on glass [8]. To combat specimen incompatibility, the glass can be coated (e.g., with poly-L-lysine, gelatin, or fibronectin) or one can use dishes constructed out of optical polymers designed to be compatible with the immersion oil [9]. To help determine what works for a specific cell type, we recommend looking in the literature at what others have used, as the substrate used can affect biological processes. Please note that standard tissue-culture plastic is autofluorescent (Box 2), so avoid regular culture dishes if possible.

The imaging media will affect both sample health and fluorescent signal. Agents such as antioxidants and ROS scavengers can be added to the media to reduce phototoxicity and photobleaching but must be tested for effects on the health of the sample [10,11]. Typical media components such as phenol red, fetal bovine serum, riboflavins and vitamins, or nutrient-rich culture media such as Luria broth (LB) can produce a high fluorescence background; thus, the components added to media must be balanced such that the sample is healthy and the signal is detectable [12–14]. It is noteworthy that imaging bacteria in a different growth media (e.g., on agarose pads made of minimal media when bacteria were grown in nutrient-rich media) may trigger stress responses that affect the localization of proteins, especially those involved in cell envelope biogenesis [15].

Choosing dyes and fluorescent proteins with longer excitation wavelengths (e.g., NucRed instead of Hoechst) and using gentler illumination settings (lower irradiation, shorter exposure times, etc.) will reduce phototoxicity and facilitate longer periods of imaging. It is always a good idea to simultaneously monitor the sample in brightfield mode during imaging, as certain morphological changes readily visible in brightfield (e.g., blebs, condensed nuclei, rounded cells) can indicate cell stress. In the case of bacteria and prokaryotes in general, phototoxicity is easily spotted as cell growth and/or division are immediately stalled. Additionally, in many bacterial cells, chromosome compaction is affected by phototoxicity and photodamage, resulting in evident changes in morphology and dynamics.

### Fixed samples

The final quality of labeling in fixed samples depends on proper fixation and permeabilization. The type of fixative, its concentration, and the fixation conditions (e.g., temperature, buffer components, and fixation length) have large effects on the preservation of the cellular structures and the general morphology of the sample. When possible, it is useful to assess how fixation affects morphology by comparing to live specimens. As with many other aspects, the fixation type and conditions should be optimized for each sample and staining. One example is immunofluorescence experiments, in which antibodies are used to label proteins of interest, where permeabilization is needed so that antibodies can access the intracellular space. It is important to note that permeabilization can remove soluble proteins and lipids that are not cross-linked, so this step must also be optimized for a given target.

The key to successful immunofluorescence labeling is to validate the antibody and to use the appropriate controls. Do not assume that antibodies have been validated to ensure specificity to the target protein. Additionally, secondary antibodies (Box 2) can bind nonspecifically to different cellular structures. It is important to take the time to validate each antibody, as the issues with antibody integrity are well known [16]. Appropriate blocking (Box 2) during immunofluorescence labeling will reduce nonspecific binding, but nonoptimal blocking conditions can increase background, so testing the effect of blocking as well as secondary-antibody-only controls are important during the optimization process. Isotype controls are also highly recommended, and when possible, knockdown and knockout controls are the gold standard for testing the specificity of the primary antibody.

In addition to immunofluorescence labeling, dyes are available that specifically label different cellular components (e.g., MitoTracker for mitochondria and DAPI for DNA, see Fig 2). When using such dyes, follow the manufacturer’s recommendations, as some do not withstand fixation. If detecting genetically expressed fluorescent proteins in a fixed sample, note that the fixative can affect the structure of the fluorescent proteins and alter their localization [17,18]. If the signal is no longer detectable after fixation, the fluorescent protein can be immunolabeled.

The last consideration for fixed samples is the mounting medium. Different mounting media are not alike and have varying compatibility with different fluorophores. Mounting media maintain the appropriate pH for the fluorophore, have photobleaching-reducing agents, and have a high RI meant to reduce aberrations due to a mismatch in RI between the sample, the coverslip, and the immersion medium. Many mounting media come with DAPI included, but these may produce high background or uneven staining in tissue sections and thus should be validated before use [19,20].

### Selecting a sample type

Cultured cells (e.g., stable cell lines, stem cell-derived, or dissociated cells) are one of the most widely used sample types to address biological processes in image-based science, especially because they are easy to maintain and observe under a microscope. However, this is an artificial system, as cells usually exist within the context of a tissue or organism. Thus, for some biological questions, more complex systems like whole tissues or 3D organoids may be more appropriate.

Whole tissue is more difficult to stain and image. Staining efficiency is affected by antigen (Box 2) accessibility and tissue penetration of the antibody or stain. Image quality is reduced by the different structures that heavily scatter light. While tissue sectioning may be an appropriate solution for imaging some thick tissues, imaging thin sections limits three-dimensional information and therefore imaging intact tissue is sometimes necessary. In this case, tissue clearing may be used to make the sample transparent and thus limit light scattering. Tissue clearing comes in many varieties and new methods are constantly being published [21]. Thus, it is a good idea to do a literature search to select a method that has been successful for a particular sample and/or target. Additionally, most clearing techniques require special objectives and/or microscope modalities and are not suitable for all imaging setups.

For live tissue, ex vivo techniques can be used [19] where an excised piece of tissue is kept alive in a chamber providing oxygenated media and laminar flow. However, this imaging requires heating, flow, and oxygenation equipment in addition to the microscope and must be carefully planned. Another alternative for imaging living cells in context is intravital imaging [22]. In most cases, surgery is used to generate “optical windows” in an organism to access the specific organ or feature of interest. Like ex vivo imaging, specific environmental conditions and equipment are required to maintain optimal imaging conditions and specialized instrumentation may also be required (e.g., multiphoton microscope). Please note, intravital imaging requires skilled veterinary technique and approval by an institutional ethics committee.

Most of the time, sample preparation is determined by the target biological question (e.g., an experiment measuring cell motility cannot be done on fixed samples), but when there are multiple options, consider techniques that will best preserve the pertinent signal, ideally with minimal changes to cell biology.

## Image acquisition

Fluorescence microscopes are available with a large number of features and capabilities [23]. Before deciding which type of microscope will best suit the experiment, consider the optical properties of the sample.

### Optical properties of the sample

As a starting point, it is important to consider the sample in terms of its transparency and thickness. Relatively thin specimens such as tissue culture cells and ultrathin cryosections (<10 μm thick) do not absorb or scatter light significantly, so they can be readily imaged using standard widefield [24] and some types of super-resolution [25,26] microscopy.

Tissue sections thicker than 10 to 20 μm absorb and scatter light more heavily, and also introduce significant out-of-focus fluorescence, leading to haziness and blur in the image. Good choices here are confocal microscopy [27] and/or computational deconvolution [28] techniques. These approaches allow acquisition of “optical sections” that enhance contrast and resolution compared to widefield techniques. If lacking a confocal microscope, a good option for samples with thicknesses up to about 20 μm is to combine widefield with deconvolution, which computationally improves the sharpness and resolution of images by removing out-of-focus light. For samples from 20 to 150 μm thick, confocal or multiphoton microscopes are the gold standard for optical sectioning.

Confocal microscopes reach their usable imaging limit at about 100 to 150 μm due to light absorption and scattering, though the maximum depth can vary depending on the optical properties of the sample. Multiphoton microscopy [29] allows imaging of thicker sections by using longer wavelength lasers to excite fluorescence. When working with fixed tissue, optical clearing [21,30] can improve tissue penetration in most fluorescent microscopy varieties. Optical clearing combined with light sheet microscopy [31] is the gold standard for whole tissue imaging. Light sheet microscopy is also useful for inherently clear samples such as fixed whole embryos from various species and for observing development of live specimens in early developmental stages.

Not every lab will have access to all types of microscopes, and it is important to remember that an experiment done on a nearly-as-good microscope is more informative than an experiment never done because the perfect microscope could not be accessed. Much can be done with a basic widefield microscope (e.g., second scenario in Fig 2); when not sufficient, other labs or institutions may allow access to the optimal microscopes needed. If required, there are also several global opportunities to access microscopes through collaborative initiatives that are working to increase accessibility (Table 1).

**Table 1:** Beginner-friendly resource list.

Resource name	Link	Brief description
**Sample preparation**		
FPbase [7]	https://www.fpbase.org/	Database for identifying fluorophores by brightness, spectra and assessing compatibility with other fluorophores and with microscope filters.
Bio-protocol	https://bio-protocol.org/en/about	Website to search for protocols across biological disciplines, including protocols associated with work published elsewhere. All protocols are available under an open access license (CC BY or CC BY-NC).
protocols.io	https://www.protocols.io/	A secure platform for developing and sharing reproducible methods.
Designing a rigorous microscopy experiment: Validating methods and avoiding bias [32]	https://rupress.org/jcb/article/218/5/1452/120908/Designing-a-rigorous-microscopy-experiment	Review of aspects of designing a rigorous light microscopy experiment, including validation of samples and imaging, identification and correction of errors, and strategies to avoid biases.
**Image acquisition**		
Nikon MicroscopyU [33]	https://www.microscopyu.com/microscopy-basics	Fundamentals of microscopy explained for beginners with lots of images and plain language descriptions of terms used in microscopy.
Fluorescence microscopy—avoiding the pitfalls [34]	https://journals.biologists.com/jcs/article/120/10/1703/29404/Fluorescence-microscopy-avoiding-the-pitfalls	Short overview of some of the most common pitfalls for beginners to fluorescence microscopy.
Best practices and tools for reporting reproducible fluorescence microscopy methods [14]	https://www.nature.com/articles/s41592-021-01156-w	Guidelines and resources for accurate reporting of the most common fluorescence light microscopy techniques, emphasizing the impact of accurate microscopy metadata on data interpretation.
Advanced Imaging Center	https://www.aicjanelia.org/apply	Access to the state-of-the-art microscopy instruments and imaging experts.
Africa Microscopy Initiative	https://www.microscopy.africa/	Access to advanced microscopes, molecular biology, and cell culture equipment for scientists in Africa.
Euro-Bioimaging	https://www.eurobioimaging.eu	Access to microcopy instruments and training for scientists in Europe.
**Image analysis**		
Image.sc [35]	https://forum.image.sc/	Discussion forum for bioimage analysis software.
Peter Bankhead’s Intro to Bioimage Analysis [36]	https://bioimagebook.github.io/	Guide for absolute beginners to image analysis, including embedded questions/answers, exercises with Python and ImageJ, and videos to check understanding.
Reproducible image handling and analysis [37]	https://www.embopress.org/doi/full/10.15252/embj.2020105889	An article reviewing major pitfalls in image handling and how to avoid them and create reproducible analysis workflows.
Made to measure: an introduction to quantification in microscopy data [38]	https://arxiv.org/abs/2302.01657 #	An article describing several common classes of measurements made in microscopy data, as well as technical factors that may affect the results.
A Hitchhiker’s guide through the bioimage analysis software universe [39]	https://febs.onlinelibrary.wiley.com/doi/full/10.1002/1873-3468.14451	An article that gives guidance and a glossary of available image analysis software and packages.
BioImage Informatics Index [40]	https://biii.eu/	Repository platform for searching bioimage analysis tools and workflows based on the problem, method, or software of choice.
iBiology Bioimage Analysis video series	https://youtu.be/1xo4vi6Ub4I	Video series that introduces bioimage analysis, including overviews of image processing, segmentation, tracking, making and interpreting measurements, tips and pitfalls.
Bioimage ANalysis Desktop (BAND)	https://band.embl.de	Access to virtual desktops allowing access to bioimage analysis software from a browser.
Galaxy Imaging Node	https://imaging.usegalaxy.eu/	A Galaxy [41] node prepopulated with a number of open-source image analysis tools and workflows, making it easy to create and share reproducible FAIR workflows.
**Data interpretation**		
Creating clear and informative image-based figures for scientific publications [42]	https://journals.plos.org/plosbiology/article?id=10.1371/journal.pbio.3001161	Review article on how to create accessible, fair scientific figures, including guidelines for microscopy images.
Modern Statistics for Modern Biology [43]	https://www.huber.embl.de/msmb/	Online statistics for biologists textbook with code examples (in R).
**Community resources**		
Global BioImaging	https://globalbioimaging.org/	Training resources, working groups, recommendations for standardization and research reproducibility for global bioimaging efforts.
Quality Assessment and Reproducibility for Instruments and Images in Light Microscopy (QUAREP–Limi) [44]	https://quarep.org/	An international association dedicated to creating standards for light microscopy as well as training materials on best practices.

### Microscope settings

Once a microscope is chosen, it is time to work out the optimal conditions for image acquisition. When acquiring images, no matter how simple or complex the microscope, take the time to understand the microscope light path from the light source to the detector(s) [33]. Knowing which components in the light path impact image quality enables accurate adjustment of their settings for the application, as well as troubleshooting any minor issues with the equipment such as incorrect filters, prisms, etc. being in the path when they should not be.

Pay particular attention to the major components that will impact the quality of the images. The following is a summary of key features and their typical adjustments to look out for. Note that while these parameters are necessary, one must also check that the microscope is in good working order (e.g., cleaned per manufacturer instructions, appropriately calibrated, objectives attached securely, brightfield light path is aligned correctly for Köhler illumination [45] (Box 2) if brightfield imaging is being used). The maintainer of the microscope will typically do this on a regular basis but speak with them about any known issues for a given microscope and how to detect and solve them.

Standard microscopes will contain a variety of components; certain components like the light source, objectives, filters, and detector (e.g., camera) are critical to good image quality. On a widefield microscope, the light source used for visual observation is often the same as for data acquisition. Advanced systems may have separate light sources for visual observation versus data acquisition. While the light path is fixed for fluorescence on most microscopes, it is often composed of several independently movable components for brightfield and must be properly aligned and calibrated before use.

It is important to choose an objective lens that has both the magnification and resolving power necessary for the experiment. While magnification is important, the resolving power of the objective is the most critical feature and is defined by the numerical aperture (or NA) and the wavelengths used for imaging. The NA is the measure of the light-gathering ability of the objective and determines how well fine features in a specimen will be resolved. Increasing NA will improve both resolution and how efficiently fluorescence emission is collected.

The color correction of the objective lens (usually labeled as Fluor, Apo, or Super Apo on the lens) is critical for multicolor imaging. It enables focusing on different colors at the same plane, allowing multiple differently colored objects to be in focus at the same time. “Fluor” typically focuses 2 colors at once, while “Apo” and “Super Apo” can handle 3 to 6 different colors. The working distance (WD, in millimeters) determines how far a lens can focus into a sample. This also includes the thickness of the coverslip and any mounting media, so be sure the WD is large enough to reach the sample. If the microscope has trouble focusing on the sample and seems to “run out” of focus, it is often because the WD of the lens is too short.

For fluorescence microscopy, one must match the filter sets to the fluorophore absorption and emission spectra. For quick reference on the spectral characteristics of different fluorophore combinations, consult a spectral viewer [7]. Using a mismatched filter can lead to flawed conclusions or even prevent imaging at all.

Most instruments use either a camera (widefield/super resolution/light sheet) or photomultiplier tube (PMT; confocal/multiphoton) for the detection of fluorescence emission. The detector converts fluorescence photons emitted from the sample to an electrical current that is then digitized and encoded as a number in the digital file that captured. Each point imaged on the sample corresponds to a pixel viewed in the final image. In applications where the goal is to detect a fluorescent reporter, the ratio of the encoded pixel value in areas with fluorescence to areas without fluorescence is often referred to as the signal-to-noise ratio; at higher signal-to-noise ratios, finer gradations in the amount of reporter present can be detected.

For cameras, adjusting the exposure controls the amount of time the camera is collecting emission light to form an image. The optimal exposure time will depend on the brightness of the sample relative to the intensity of the excitation light. Dimmer specimens require either higher intensity excitation light or longer exposure times. Standard practice is to adjust the excitation intensity to expose the specimen to the lowest dose of excitation light possible to minimize photobleaching and phototoxicity. When using fluorescence, fluorophores often photobleach easily, so it is better in general to use a lower illumination power and longer camera exposure when using camera-based systems. Exposure time is particularly important for live-cell experiments where many images are acquired over time to capture a dynamic process. Longer exposure times will also increase the overall image acquisition time. If long exposure times are needed, the field of view, or size of the region being imaged, can be reduced to produce a smaller image and minimize acquisition time. For scanning systems that use PMTs, consider line (or frame) averaging to improve the signal-to-noise ratio. Overexposure, also known as saturation, can be caused by excitation light that is too intense or exposure times that are too long and will render images unsuitable for quantification of intensity.

In conclusion, the microscopy image is more than a picture; it is a dataset that must be as high quality and reproducible as possible. It is often necessary to balance competing needs for spatial resolution, number of targets, and, when examining dynamic processes, acquisition speed. Optimization by definition is iterative, and we recommend testing out different settings before finalizing. Review and analyze preliminary data before finalizing an approach for the whole project. Finally, the imaging parameters of the microscope used must be recorded and reproduced within each experiment [14].

## Image analysis

### Common image analysis methods

Microscopy images are ultimately composed of digital numbers, which makes them a very powerful data source if used correctly. What we typically think of as pictures are grids of pixels, each with a brightness (intensity) represented by a number. As a biologist, image analysis allows for the translation of these numbers into insights that can answer biological questions. Example questions include:

1. Are cells in this treatment expressing more or less protein than controls?
2. Does the subcellular localization of this protein change in different treatments?
3. How many spots (e.g., mRNA molecules) are in each cell?
4. How do these cells move, divide, or change over time?

While some biological questions can be solved with whole image measurements, many biological questions, including all the above, require measuring individual “objects” within images. Objects can be cells or anything that can be measured individually in the image (e.g., tissue sections on a slide, spots within cells, fluorescent beads, bacteria). Sometimes, this measurement is as simple as counting objects present in an image. Identifying the presence of an object (e.g., by finding its center, but not delineating its borders) is referred to as detection. For other workflows, the number of objects alone is insufficient and other measurements about those objects are needed, such as the diameter of cells under different drug treatments. In this case, a further process known as segmentation is likely necessary. Segmentation refers to assigning the pixels in an image to different objects. Segmentation typically involves thresholding the image by deciding that pixels with intensities above a certain level belong to objects of interest and the rest are background. We focus on segmentation here as it is a common process in many bioimage analysis workflows, although new approaches with deep learning and machine learning are also being developed that can extract meaningful features without requiring delineated cell boundaries [46].

In practice, segmentation can be challenging and a simple threshold of the original image is rarely sufficient. Image processing is a category of operations that filter or transform an image to either correct for aberrations or make segmentation easier (e.g., noise suppression). The end goal of this processing and segmentation is to create a mask of objects, a map where pixels are labeled according to which object they belong (see Fig 2 for an example). A common source of confusion for beginners is what is “allowed” when processing images. In general, anything that is reproducible and produces more accurate segmentation is fair game. There is some interpretation involved in defining “accurate” segmentation, but as a good practice, we recommend overlaying outlines of identified objects on the original images to help assess segmentation accuracy.

Images can have distortions due to microscope optics and illumination sources that can be corrected with image processing. Objective lenses can cause images to have a brighter center due to spherical aberration (Box 2) and other processes. Additionally, many things in the light path can have autofluorescence that will increase the background intensity. These distortions can be corrected using illumination correction and background correction. Some microscopy systems can apply a correction during the imaging process but others require correction after, which can be part of the image analysis workflow.

When measuring the intensities of pixels belonging to objects, be sure to do so on the original images or the corrected images if background correction or illumination correction are used, and not on otherwise-processed images that may have been used for segmentation. Intensity measurements may not be required if the question of interest is better answered using shape descriptors and/or the number of objects alone; when using shape descriptors, it is critical to ensure that the spatial calibration (pixel size) is correct.

The image processing, segmentation, and measurement steps may be combined in what is often called an image analysis “pipeline” or “workflow.” A typical pipeline might begin with general steps for image processing: de-noising, illumination or background correction, and finally enhancing the corrected images to better detect the objects of interest. The resulting processed images are used to segment the objects of interest. Finally, the object locations and boundaries can be used to measure object intensity on the corrected images.

The specifics of the image analysis pipeline as well as the relevant measurements will vary depending on the sample, microscopy, and biological question posed. We present 2 specific examples of common fluorescence microscopy image analysis workflows (Fig 2) and a list of additional resources (Table 1). The accompanying companion website also includes detailed explanations of some of the common image processing operations and discussion of common pitfalls.

### Software

Once the biological question has been defined and the type of analysis decided, the next step is to select appropriate image analysis software. There are many options to pick from; we have narrowed down those discussed here to free, open-source software that have a high degree of interoperability (e.g., file types, outputs) with other software. It was also important to pick software with good documentation, including manuals and tutorials, and a history of user support. Finally, we focus on software that is easy to use, allows users to create reproducible analyses, and can process a large quantity of images. We summarize a few popular image analysis software solutions that meet these criteria below.

ImageJ is a tried and true basic image analysis software that can be extended further with the use of plugins or the “batteries-included” distribution Fiji [47–49]. CellProfiler allows users to create image analysis pipelines that are easily scalable to use for thousands of images [50]. Icy is not only an image analysis software but also a platform for exchange of protocols within the image analysis community [51]. For histopathology applications, QuPath is a tool optimized for handling large pyramidal 2D images, like those generated from slide scanners [52]. For easy-to-use machine learning pixel classification, ilastik allows users to segment, classify, and analyze their images [53]. Finally, napari is a highly supported image analysis tool that allows non-programming users to use data analysis tools written in Python [54].

Extensive curated lists of other open-source software can be found in the image analysis section of Table 1. No matter the software chosen, make sure to report any tools used, including version numbers where available. Also check for how the authors wish their software to be referenced, which often includes citing a specific publication. This is particularly important for open-source tools because the authors typically depend on grant funding to continue maintaining and advancing the software, and citations are a critical metric for obtaining funding [55].

### Data management and sharing

Recent advances in microscopy such as whole-brain imaging or sophisticated computer-controlled imaging experiments can easily generate an amount of data in the terabyte range. Traditional methods of storing and retrieving data are no longer a viable option. Furthermore, funding agencies are now integrating data management plans into the requirements for a successful grant application. The reproducibility crisis [56,57] has spawned community-led initiatives to tackle the problems that come along with big data. FAIR (Findable, Accessible, Interoperable, and Reproducible) principles are no longer a fringe initiative, but are part of the default research vocabulary [58]. Groups like QUAREP-LiMi [44] have emerged to provide guidelines for image formatting, data availability, and reporting image analysis workflows, to increase the adoption of FAIR principles in published microscopy images and data [59]. Two notable actively maintained platforms for data management are OMERO [60] and OpenBIS [61]. OMERO emphasizes image data management and sharing. OpenBIS aspires to be a comprehensive platform for scientific data, including reagent inventories, lab notebooks, and experimental data repositories.

When properly annotated with metadata of how it was created, bioimages can be a long-term resource for the scientific community that may be re-interpreted or re-analyzed with different goals in mind. While currently image data in papers is often not freely available but instead “available upon request,” sharing one’s image data can lead to new insights beyond what was conceived of in the original project. Several repositories (such as Zenodo [62] or figshare) and dedicated image archives (such as EMBL-EBI BioImage Archive [63] or Image Data Resource [64]), as well as a number of government agencies (e.g., the National Institute of Mental Health Data Archive (NDA) and the National Cancer Institute’s The Cancer Genome Atlas (TGCA) [65]), allow scientists to make image data available long term. In addition, dedicated annotated (added-value) databases are beginning to emerge that contain curated data and images to be further analyzed [66,67].

With regards to data management, using version control is critical for large datasets and complex analysis pipelines but good practice at any data size. Version control is the ability to keep track of changes to a script or document and switch to different versions at any time without losing the others. Version control is used extensively in the software industry and in code repositories such as GitHub, but it can also be found in cloud document editing (e.g., Google Docs), where versions are kept automatically. One interesting tool for version control that is popular in neuroimaging is Datalad [68]. Built on top of established version control software (Git), it provides an easy means to track changes to entire datasets, rather than only code.

## Data interpretation

Image data can support scientific observations both qualitatively (figures) and quantitatively (number tables and plots) and at times the same image data may be reused for either direction. Scientists should familiarize themselves with the distinction between quantitative and qualitative data analysis as requirements for image formats and image handling differ. Hereafter, we discuss important aspects about the presentation and interpretation of these 2 kinds of output.

### Qualitative data: Image figures

Image figures show a representative observation, a qualitative finding, or a range of possible states. Image figures primarily serve as a qualitative assessment of the data, and as such, no quantifications should be performed on images prepared for presentation purposes. To this end, consider presenting the images in “adequate resolution” that is suitable to support the claim. Precise pixel sizes necessarily depend on the image acquisition method, object size, and experimental objective used. Image figures require a detailed method section (sample preparation, imaging setup, etc. [69]) and a figure legend briefly explaining image object (specimen, tissue, cell type), colors (fluorophores, stains, dyes), and all annotations [59].

When adjusting images for presentation, it is acceptable to adjust the image size, orientation, and the frame (crop). Note that depending on the software used, pixel information may be redistributed when images are rotated. All formatting adjustments that do not change the conclusions are allowed, provided one is familiar with instances that would be considered misleading [70]. For example, adjustments of the brightness and contrast are permitted when they are required to display observations faithfully; adjustments that remove image details or scale intensity nonlinearly are generally considered misleading [70,71].

To allow broad accessibility, authors should carefully choose the colors used in images. Fluorescence microscope image data is usually collected in grayscale and may be presented as such to best present image details (see Fig 2 for an example). Often, fluorescent channels are shown in the color representing the capturing wavelength (e.g., blue, green, red); however, authors should be aware that color-coding intensity values on a black background can reduce the level of detail depending on the color used [42]. When multicolor images are shown, consider whether color combinations are accessible to color-blind audiences. For example, do not combine red with green, but rather use magenta and green, (see [42] and Fig 2 for examples) and possibly additionally show individual channels in grayscale to maximize accessibility and visual detail. Tools for color blindness simulation exist in image processing software (ImageJ/Fiji) and visibility of colors in final image figures can be tested with applications such as ColorOracle [72].

Interpretable images must contain at minimum an indication of the physical size of the object shown, most often done with a scale bar. Unfortunately, scale information is missing or incomplete in almost 50% of biology publications [42]. Annotations added to images should be explained in the figure or in the figure legend. Annotations may include symbols, arrows, letter codes, regions of interest, and zoomed insets. Annotations are almost never useful in the abstract, but must be paired in the figure or its legend with an explanation of all colors and symbols used within the figure to be fully interpreted.

### Quantitative data: Measurements and statistics

Quantitative assessment of images begins with ensuring that all images to be analyzed are suitable for meaningful comparison. Measurements from images are affected not only by biological phenomena that are of interest to the researcher, but also by confounding variables (e.g., the amount of time each plate sat at room temperature before imaging) [38,73]. Mitigating these effects starts with keeping track of metadata about the experimental and imaging conditions [74] and carefully designing the sample preparation and microscopy to minimize differences between samples destined for quantitative comparison.

Statistics provides a formal way to utilize measurements extracted from images in order to answer a specific question. The path towards identifying an appropriate statistical analysis is composed of 3 steps: (1) formulate a clear question; (2) identify a statistical tool (metric, test) that can answer that question; and (3) understand the underlying assumptions of that tool and whether the measurements considered satisfy these assumptions. While a full treatment of statistics is beyond the scope of this Essay, a few common questions recur.

The first task is typically to determine which metric(s) should be used. It can often be complicated to decide between a host of similar metrics to find the best match for the biological question; the more specific the question, the greater the chance of success. “Did expression of marker X change in these cells?”, for example, could be answered by looking at the total intensity of a given fluorophore, its mean intensity, its distribution across certain parts of the cell, or via many other metrics as well; “Did the total amount of marker X increase within the nucleus?” is easier to use for metric selection.

Once a metric is selected, it is typically compared statistically across a number of conditions; while many statistical tools assume that the data/measurements considered follow a Gaussian/normal distribution, this is rarely the case in biological data [75]. Pixel values in microscopy images are most often composed of different groups (e.g., background and foreground intensities, positive and negative phenotype), resulting in multimodal distributions of measurements. The validity of the assumption of normality can easily be verified relying on tests such as the Kolmogorov–Smirnov [76] or the Shapiro–Wilk [77] tests.

The statistical considerations listed here are crucial to consider before data collection has been completed, as the number of images acquired at the microscope is best chosen based on the number of samples required to detect an effect of interest. How many independent samples are required, however, depends on the statistical analysis being performed and on an assumed statistical model of the studied dataset, such as how large of an effect size is expected between conditions and the amount of technical variability that will be seen. Pilot experiments and pilot analyses of candidate metrics are critical in estimating these factors; estimates allow the use of power analysis [78] to empirically determine the number of samples that must be acquired in each replicate of the final experiment. Further complicating the issue of sample number, what is considered as “an individual sample” depends on the experimental question. Depending on the information being sought, individual samples may be biological replicates (e.g., animals), technical replicates (e.g., individual Petri dishes or wells), or individual cells. In calculating and reporting summary statistics, one must be explicit about what level of data is being compared; where possible, consider displaying quantitative data using approaches that display summary statistics (e.g., the mean) alongside the individual data points (see Fig 2 for an example), such as demonstrated with SuperPlots [79]. Resources in Table 1 dig further into these questions and provide deeper insights into the most common statistical tools used in biological research and/or bioimaging.

## Conclusions

The success or failure of a microscopy experiment ultimately rests upon the decisions made in its design and execution; here, we have presented general guidelines and basic concepts that can guide the decision-making process when designing one’s experiments. Designing experiments so that they can be quantitative and reproducible not only benefits the experimental scientist but also the wider community. To that end, we strongly recommend that data, analysis pipelines, and other resources be made open source and freely available to the public. The information presented here is not exhaustive and we further recommend consulting with experts, exploring resources linked here and on the companion website, and reading relevant published literature and protocols. Regional organizations dedicated to bioimaging and/or bioimage analysis are present around the globe and are often in an excellent position to connect beginners with local resources available to them (Table 2).

**Table 2:** Resources for beginners in different regions.

Region specific community resources	
BioImaging North America (BINA)	https://www.bioimagingnorthamerica.org/
Latin America BioImaging	https://www.latambioimaging.org/
African BioImaging Consortium	https://www.africanbioimaging.org/
South Africa BioImaging	https://www.sabioimaging.org/
Euro-bioimaging	https://www.eurobioimaging.eu/
Advanced BioImaging support (Japan)	https://www.nibb.ac.jp/abis/
Microscopy Australia	https://micro.org.au/
Canada BioImaging	https://www.canadabioimaging.org/
Singapore Microscopy Infrastructure Network	https://www.singascope.sg/

Finally, the bioimaging and bioimage analysis communities have in recent years created rich online spaces that can be accessed via the forums image.sc [35] and μforum [80]. On these forums, members can pose questions to imaging and image analysis experts, ranging from issues with specific software to general experimental design questions and questions about controls. These forums foster an inclusive, friendly environment for beginners and experts alike, with the goal of making expert advice more accessible, especially for scientists at institutions without a dedicated imaging or image analysis core facility. While many factors go into the design of a bioimaging experiment, with education and advice on critical decisions, one can procure the best chance at success.

## Abbreviations

DIC: differential interference contrast
LB: Luria broth
NA: numerical aperture
PMT: photomultiplier tube
RI: refractive index
ROS: reactive oxygen species
WD: working distance

## References

[1] Carpenter AE, Jones TR, Lamprecht MR, Clarke C, Kang I, Friman O, et al. Genome Biology. 2006. R100. doi: 10.1186/gb-2006-7-10-r100 PMC1794559 17076895

[2] Ljosa V, Sokolnicki KL, Carpenter AE. Annotated high-throughput microscopy image sets for validation. Nat Methods. 2012;9:637. doi: 10.1038/nmeth.2083 22743765 PMC3627348

[3] Executable Books Community. Jupyter Book 2020. doi: 10.5281/zenodo.4539666

[4] Waters JC, Wittmann T. Chapter 1—Concepts in quantitative fluorescence microscopy. In: Waters JC, Wittman T, editors. Methods in Cell Biology. Academic Press; 2014. p. 1–18. 10.1016/B978-0-12-420138-5.00001-X 24974019

[5] Keppler A, Pick H, Arrivoli C, Vogel H, Johnsson K. Labeling of fusion proteins with synthetic fluorophores in live cells. Proc Natl Acad Sci U S A. 2004;101:9955–9959. doi: 10.1073/pnas.0401923101 15226507 PMC454197

[6] Los GV, Encell LP, McDougall MG, Hartzell DD, Karassina N, Zimprich C, et al. HaloTag: a novel protein labeling technology for cell imaging and protein analysis. ACS Chem Biol. 2008;3:373–382. doi: 10.1021/cb800025k 18533659

[7] Lambert TJ. FPbase: a community-editable fluorescent protein database. Nat Methods. 2019;16:277–278. doi: 10.1038/s41592-019-0352-8 30886412

[8] Scholz WK. Cell Adhesion and Growth on Coated or Modified Glass or Plastic Surfaces. 2010 [cited 2023 Mar 20]. Available from: https://www.semanticscholar.org/paper/2a0b01226c933f4dcb73b550d65e0238264493cd.

[9] Compatible Immersion Oil for Polymer Coverslip- FAQ—ibidi. [cited 2022 Dec 6]. Available from: https://ibidi.com/content/551-immersion-oils-compatible-with-ibidi-labware-products.

[10] Icha J, Weber M, Waters JC, Norden C. Phototoxicity in live fluorescence microscopy, and how to avoid it. Bioessays. 2017:39. doi: 10.1002/bies.201700003 28749075

[11] Tosheva KL, Yuan Y, Matos Pereira P, Culley S, Henriques R. Between life and death: strategies to reduce phototoxicity in super-resolution microscopy. J Phys D Appl Phys. 2020;53:163001. doi: 10.1088/1361-6463/ab6b95 33994582 PMC8114953

[12] Frigault MM, Lacoste J, Swift JL, Brown CM. Live-cell microscopy–tips and tools. J Cell Sci. 2009:753–767. doi: 10.1242/jcs.033837 19261845

[13] Bogdanov AM, Kudryavtseva EI, Lukyanov KA. Anti-fading media for live cell GFP imaging. PLoS ONE. 2012;7:e53004. doi: 10.1371/journal.pone.0053004 23285248 PMC3528736

[14] Montero Llopis P, Senft RA, Ross-Elliott TJ, Stephansky R, Keeley DP, Koshar P, et al. Best practices and tools for reporting reproducible fluorescence microscopy methods. Nat Methods. 2021;18:1463–1476. doi: 10.1038/s41592-021-01156-w 34099930

[15] Hocking J, Priyadarshini R, Takacs CN, Costa T, Dye NA, Shapiro L, et al. Osmolality-dependent relocation of penicillin-binding protein PBP2 to the division site in Caulobacter crescentus. J Bacteriol. 2012;194:3116–3127. doi: 10.1128/JB.00260-12 22505677 PMC3370875

[16] Uhlen M, Bandrowski A, Carr S, Edwards A, Ellenberg J, Lundberg E, et al. A proposal for validation of antibodies. Nat Methods. 2016;13:823–827. doi: 10.1038/nmeth.3995 27595404 PMC10335836

[17] Irgen-Gioro S, Yoshida S, Walling V, Chong S. Fixation can change the appearance of phase separation in living cells. Elife. 2022:11. doi: 10.7554/eLife.79903 36444977 PMC9817179

[18] Li M-W, Zhou L, Lam H-M. Paraformaldehyde Fixation May Lead to Misinterpretation of the Subcellular Localization of Plant High Mobility Group Box Proteins. PLoS ONE. 2015;10:e0135033. doi: 10.1371/journal.pone.0135033 26270959 PMC4535772

[19] Krishnamurthy S, Brown JQ, Iftimia N, Levenson RM, Rajadhyaksha M. Ex Vivo Microscopy: A Promising Next-Generation Digital Microscopy Tool for Surgical Pathology Practice. Arch Pathol Lab Med. 2019;143:1058–1068. doi: 10.5858/arpa.2019-0058-RA 31295016 PMC7365575

[20] Quantitative Imaging in Cell Biology. Methods Cell Biol. 2014. doi: 10.1016/c2013-0-09942-2 24974047

[21] Richardson DS, Guan W, Matsumoto K, Pan C, Chung K, Ertürk A, et al. TISSUE CLEARING. Nat Rev Methods Primers. 2021:1. doi: 10.1038/s43586-021-00080-9 35128463 PMC8815095

[22] Pittet MJ, Weissleder R. Intravital imaging. Cell. 2011;147:983–991. doi: 10.1016/j.cell.2011.11.004 22118457 PMC3824153

[23] Sanderson MJ, Smith I, Parker I, Bootman MD. Fluorescence microscopy. Cold Spring Harb Protoc. 2014; db.top071795. doi: 10.1101/pdb.top071795 25275114 PMC4711767

[24] Swedlow JR, Platani M. Live cell imaging using wide-field microscopy and deconvolution. Cell Struct Funct. 2002;27:335–341. doi: 10.1247/csf.27.335 12502887

[25] Verdaasdonk JS, Stephens AD, Haase J, Bloom K. Bending the rules: widefield microscopy and the Abbe limit of resolution. J Cell Physiol. 2014;229:132–138. doi: 10.1002/jcp.24439 23893718 PMC4076117

[26] Stemmer A, Beck M, Fiolka R. Widefield fluorescence microscopy with extended resolution. Histochem Cell Biol. 2008;130:807–817. doi: 10.1007/s00418-008-0506-8 18810482

[27] Jonkman J, Brown CM, Wright GD, Anderson KI, North AJ. Tutorial: guidance for quantitative confocal microscopy. Nat Protoc. 2020;15:1585–1611. doi: 10.1038/s41596-020-0313-9 32235926

[28] Swedlow JR. Quantitative Fluorescence Microscopy and Image Deconvolution. Methods in Cell Biology. Academic Press; 2007. p. 447–465. 10.1016/S0091-679X(06)81021-6 17519179

[29] Ustione A, Piston DW. A simple introduction to multiphoton microscopy. J Microsc. 2011;243:221–226. doi: 10.1111/j.1365-2818.2011.03532.x 21777244

[30] Gómez-Gaviro MV, Sanderson D, Ripoll J, Desco M. Biomedical Applications of Tissue Clearing and Three-Dimensional Imaging in Health and Disease. iScience. 2020;23:101432. doi: 10.1016/j.isci.2020.101432 32805648 PMC7452225

[31] Wan Y, McDole K, Keller PJ. Light-Sheet Microscopy and Its Potential for Understanding Developmental Processes. Annu Rev Cell Dev Biol. 2019;35:655–681. doi: 10.1146/annurev-cellbio-100818-125311 31299171

[32] Jost AP-T, Waters JC. Designing a rigorous microscopy experiment: Validating methods and avoiding bias. J Cell Biol. 2019;218:1452–1466. doi: 10.1083/jcb.201812109 30894402 PMC6504886

[33] Spring KR, Komatsu H, Scott ML, Schwartz SA, Fellers TJ, Carr KE. et al. Basic Concepts and Formulas in Microscopy. In: Nikon’s MicroscopyU [Internet]. Available from: https://www.microscopyu.com/microscopy-basics.

[34] Brown CM. Fluorescence microscopy—avoiding the pitfalls. J Cell Sci. 2007;120:1703–1705. doi: 10.1242/jcs.03433 17502480

[35] Rueden CT, Ackerman J, Arena ET, Eglinger J, Cimini BA, Goodman A, et al. Scientific Community Image Forum: A discussion forum for scientific image software. PLoS Biol. 2019;17:e3000340. doi: 10.1371/journal.pbio.3000340 31216269 PMC6602289

[36] Bankhead P. Introduction to Bioimage Analysis. 2022. Available from: https://bioimagebook.github.io/README.html.

[37] Miura K, Nørrelykke SF. Reproducible image handling and analysis. EMBO J. 2021;40:e105889. doi: 10.15252/embj.2020105889 33480052 PMC7849301

[38] Culley S, Caballero AC, Burden JJ, Uhlmann V. Made to measure: an introduction to quantification in microscopy data. arXiv [q-bioQM]. 2023. Available from: http://arxiv.org/abs/2302.01657. 10.1111/jmi.13208 37269048

[39] Haase R, Fazeli E, Legland D, Doube M, Culley S, Belevich I, et al. A Hitchhiker’s guide through the bio-image analysis software universe. FEBS Lett. 2022;596:2472–2485. doi: 10.1002/1873-3468.14451 35833863

[40] Paul-Gilloteaux P, Tosi S, Hériché J-K, Gaignard A, Ménager H, Marée R, et al. Bioimage analysis workflows: community resources to navigate through a complex ecosystem. F1000Res. 2021;10:320. doi: 10.12688/f1000research.52569.1 34136134 PMC8182692

[41] Community Galaxy. The Galaxy platform for accessible, reproducible and collaborative biomedical analyses: 2022 update. Nucleic Acids Res. 2022;50:W345–W351. doi: 10.1093/nar/gkac247 35446428 PMC9252830

[42] Jambor H, Antonietti A, Alicea B, Audisio TL, Auer S, Bhardwaj V, et al. Creating clear and informative image-based figures for scientific publications. PLoS Biol. 2021;19:e3001161. doi: 10.1371/journal.pbio.3001161 33788834 PMC8041175

[43] Holmes S, Huber W. Modern Statistics for Modern Biology. Feb 2019. Available from: https://www.huber.embl.de/msmb/.

[44] Nelson G, Boehm U, Bagley S, Bajcsy P, Bischof J, Brown CM, et al. QUAREP-LiMi: A community-driven initiative to establish guidelines for quality assessment and reproducibility for instruments and images in light microscopy. arXiv [q-bioOT]. 2021. Available from: http://arxiv.org/abs/2101.09153. 10.1111/jmi.13041 PMC10388377 34214188

[45] Köhler A. New Method of Illumination for Photomicrographical Purposes. J R Microsc Soc. 1894;14:261–262.

[46] Lucas AM, Ryder PV, Li B, Cimini BA, Eliceiri KW, Carpenter AE. Open-source deep-learning software for bioimage segmentation. Mol Biol Cell. 2021;32:823–829. doi: 10.1091/mbc.E20-10-0660 33872058 PMC8108523

[47] Schneider CA, Rasband WS, Eliceiri KW. NIH Image to ImageJ: 25 years of image analysis. Nat Methods. 2012;9:671–675. doi: 10.1038/nmeth.2089 22930834 PMC5554542

[48] Schindelin J, Arganda-Carreras I, Frise E, Kaynig V, Longair M, Pietzsch T, et al. Fiji: an open-source platform for biological-image analysis. Nat Methods. 2012;9:676–682. doi: 10.1038/nmeth.2019 22743772 PMC3855844

[49] Rueden CT, Schindelin J, Hiner MC, DeZonia BE, Walter AE, Arena ET, et al. ImageJ2: ImageJ for the next generation of scientific image data. BMC Bioinformatics. 2017;18:529. doi: 10.1186/s12859-017-1934-z 29187165 PMC5708080

[50] Stirling DR, Swain-Bowden MJ, Lucas AM, Carpenter AE, Cimini BA, Goodman A. CellProfiler 4: improvements in speed, utility and usability. BMC Bioinformatics. 2021;22:433. doi: 10.1186/s12859-021-04344-9 34507520 PMC8431850

[51] de Chaumont F, Dallongeville S, Chenouard N, Hervé N, Pop S, Provoost T, et al. Icy: an open bioimage informatics platform for extended reproducible research. Nat Methods. 2012;9:690–696. doi: 10.1038/nmeth.2075 22743774

[52] Bankhead P, Loughrey MB, Fernández JA, Dombrowski Y, McArt DG, Dunne PD, et al. QuPath: Open source software for digital pathology image analysis. Sci Rep. 2017;7:16878. doi: 10.1038/s41598-017-17204-5 29203879 PMC5715110

[53] Berg S, Kutra D, Kroeger T, Straehle CN, Kausler BX, Haubold C, et al. ilastik: interactive machine learning for (bio)image analysis. Nat Methods. 2019;16:1226–1232. doi: 10.1038/s41592-019-0582-9 31570887

[54] Sofroniew N, Lambert T, Evans K, Nunez-Iglesias J, Bokota G, Winston P, et al. napari: a multi-dimensional image viewer for Python. 2022. doi: 10.5281/zenodo.7276432

[55] Levet F, Carpenter AE, Eliceiri KW, Kreshuk A, Bankhead P, Haase R. Developing open-source software for bioimage analysis: opportunities and challenges. F1000Res. 2021;10:302. doi: 10.12688/f1000research.52531.1 34249339 PMC8226416

[56] Freedman LP, Cockburn IM, Simcoe TS. The Economics of Reproducibility in Preclinical Research. PLoS Biol. 2015;13:e1002165. doi: 10.1371/journal.pbio.1002165 26057340 PMC4461318

[57] Baker M. 1,500 scientists lift the lid on reproducibility. In: Nature Publishing Group UK [Internet]. 25 May 2016 [cited 2023 Mar 28]. doi: 10.1038/533452a 27225100

[58] Wilkinson MD, Dumontier M, Aalbersberg IJJ, Appleton G, Axton M, Baak A, et al. The FAIR Guiding Principles for scientific data management and stewardship. Sci Data. 2016;3:160018. doi: 10.1038/sdata.2016.18 26978244 PMC4792175

[59] Schmied C, Nelson M, Avilov S, Bakker G-J, Bertocchi C, Bischof J, et al. Community-developed checklists for publishing images and image analysis. arXiv [q-bioOT]. 2023. Available from: http://arxiv.org/abs/2302.07005. 10.1038/s41592-023-01987-9 PMC10922596 37710020

[60] Allan C, Burel J-M, Moore J, Blackburn C, Linkert M, Loynton S, et al. OMERO: flexible, model-driven data management for experimental biology. Nat Methods. 2012;9:245–253. doi: 10.1038/nmeth.1896 22373911 PMC3437820

[61] Bauch A, Adamczyk I, Buczek P, Elmer F-J, Enimanev K, Glyzewski P, et al. openBIS: a flexible framework for managing and analyzing complex data in biology research. BMC Bioinformatics. 2011;12:468. doi: 10.1186/1471-2105-12-468 22151573 PMC3275639

[62] European Organization for Nuclear Research, OpenAIRE. Zenodo. CERN. 2013. doi: 10.25495/7GXK-RD71 PMC3635800 23549990

[63] Hartley M, Kleywegt GJ, Patwardhan A, Sarkans U, Swedlow JR, Brazma A. The BioImage Archive—Building a Home for Life-Sciences Microscopy Data. J Mol Biol. 2022;434:167505. doi: 10.1016/j.jmb.2022.167505 35189131

[64] Williams E, Moore J, Li SW, Rustici G, Tarkowska A, Chessel A, et al. The Image Data Resource: A Bioimage Data Integration and Publication Platform. Nat Methods. 2017;14:775–781. doi: 10.1038/nmeth.4326 28775673 PMC5536224

[65] Cancer Genome Atlas Research Network, Weinstein JN, Collisson EA, Mills GB, Shaw KRM, Ozenberger BA, et al. The Cancer Genome Atlas Pan-Cancer analysis project. Nat Genet. 2013;45:1113–1120. doi: 10.1038/ng.2764 24071849 PMC3919969

[66] Ellenberg J, Swedlow JR, Barlow M, Cook CE, Sarkans U, Patwardhan A, et al. A call for public archives for biological image data. Nat Methods. 2018;15:849–854. doi: 10.1038/s41592-018-0195-8 30377375 PMC6884425

[67] OSF. [cited 2022 Dec 14]. Available from: https://osf.io/.

[68] Halchenko Y, Meyer K, Poldrack B, Solanky D, Wagner A, Gors J, et al. DataLad: distributed system for joint management of code, data, and their relationship. J Open Source Softw. 2021;6:3262. 10.21105/joss.03262 PMC11514317 39469147

[69] Marqués G, Pengo T, Sanders MA. Imaging methods are vastly underreported in biomedical research. Elife. 2020:9. doi: 10.7554/eLife.55133 32780019 PMC7434332

[70] Cromey DW. Avoiding twisted pixels: ethical guidelines for the appropriate use and manipulation of scientific digital images. Sci Eng Ethics. 2010;16:639–667. doi: 10.1007/s11948-010-9201-y 20567932 PMC4114110

[71] Rossner M, Yamada KM. What’s in a picture? The temptation of image manipulation. J Cell Biol. 2004;166:11–15. doi: 10.1083/jcb.200406019 15240566 PMC2172141

[72] Jenny B, Kelso NV. Color Design for the Color Vision Impaired. CPJ. 2007:61–67.

[73] Soneson C, Gerster S, Delorenzi M. Batch effect confounding leads to strong bias in performance estimates obtained by cross-validation. PLoS ONE. 2014;9:e100335. doi: 10.1371/journal.pone.0100335 24967636 PMC4072626

[74] Caicedo JC, Cooper S, Heigwer F, Warchal S, Qiu P, Molnar C, et al. Data-analysis strategies for image-based cell profiling. Nat Methods. 2017;14:849–863. doi: 10.1038/nmeth.4397 28858338 PMC6871000

[75] Fay DS, Gerow K. A biologist’s guide to statistical thinking and analysis. WormBook. 2018. 10.1895/wormbook.1.159.1 PMC3880567 23908055

[76] Kolmogorov AN. Sulla determinazione empirica di una lgge di distribuzione. G Ist Ital Attuari. 1933;4:83–91.

[77] Shapiro SS, Wilk MB. An analysis of variance test for normality (complete samples). Biometrika. 1965;52:591–611.

[78] Neyman J, Pearson ES. ON THE USE AND INTERPRETATION OF CERTAIN TEST CRITERIA FOR PURPOSES OF STATISTICAL INFERENCE. Biometrika. 1928;20A:263–294.

[79] Lord SJ, Velle KB, Mullins RD, Fritz-Laylin LK. SuperPlots: Communicating reproducibility and variability in cell biology. J Cell Biol. 2020:219. doi: 10.1083/jcb.202001064 32346721 PMC7265319

[80] Microforum. In: Microforum [Internet]. [cited 2023 May 15]. Available from: https://forum.microlist.org/.

